# Ulinastatin in the Management of Severe Acute Alcoholic Pancreatitis: A Case Series

**DOI:** 10.31729/jnma.4987

**Published:** 2021-12-31

**Authors:** Niraj Kumar Keyal, Amit Singh, Abishek Pokhrel, Amid Bhujel, Rupesh Kumar Chaurasia

**Affiliations:** 1 Department of Critical Care Medicine, B &C Medical College Teaching Hospital & Research center, Birtamod, Jhapa, Nepal; 2 Department of General Surgery, B &C Medical College Teaching Hospital & Research center, Birtamod, Jhapa, Nepal; 3 Department of Radiology, B &C Medical College Teaching Hospital & Research Center, Birtamod, Jhapa, Nepal

**Keywords:** *alcoholic*, *antiproteases*, *pancreatitis*

## Abstract

Severe acute alcoholic pancreatitis is a second common form of pancreatitis that requires intensive care unit care and has high morbidity and mortality due to lacking specific treatment. Management of alcoholic pancreatitis is generally non-specific and supportive. We hereby present a case-series of three patients that describes the successful treatment of severe acute alcoholic pancreatitis with ulinastatin and other supportive treatment. From this we want to emphasize that ulinastatin a protease inhibitor can be used in the treatment of alcoholic pancreatitis.

## INTRODUCTION

The diagnosis of alcoholic pancreatitis (AP) should be entertained when a patient has consumed alcohol greater than 50 grams per day over 5 years.^1^ The incidence of alcoholic pancreatitis ranges from 22-57% and mortality in severe pancreatitis is 30-80%.^2^

The mortality in the first week is due to massive inflammatory responses leading to multi-organ failure and in a later course is due to septic complications related to infection or necrosis leading to multi-organ failure. Ulinastatin is a serine protease inhibitor that can decrease inflammation and can decrease morbidity and mortality. This is the case series that has used ulinastatin in acute alcoholic pancreatitis.

## CASE REPORT

### CASE 1

A 56-year-old male, with a history of type 2 diabetes mellitus under irregular medication and daily consumer of two bottles of alcohol presented at the Emergency department of a general super specialty hospital, Birtamod, Nepal. He presented with a history of epigastric abdominal pain radiating to back, 9/10 in severity, aggravated by coughing and relieved by leaning forward and multiple episodes of non-projectile vomiting containing food particles. He also complained of high-grade fever and productive cough for 5 days.

At the time of admission to the emergency department his GCS was 15/15, pulse rate 140 beats per min, blood pressure 80/60mm Hg, respiratory rate 30/min and oxygenation saturation was 94% on 10 liters of oxygen. The chest examination showed crepitations in the right lower zone. Abdominal examination showed tenderness, guarding and rigidity at the epigastric region. Arterial blood gas analysis showed high anion gap metabolic acidosis and lactic acidosis.

An emergency physician and paramedics immediately resuscitated him by inserting 16 gauze cannula, central venous catheter and giving two liters of balanced salt solution Kabilyte (Fresenius Kabi, Pune, India) and noradrenalin was started at 0.2μg/kg/min.

**Figure 1:**
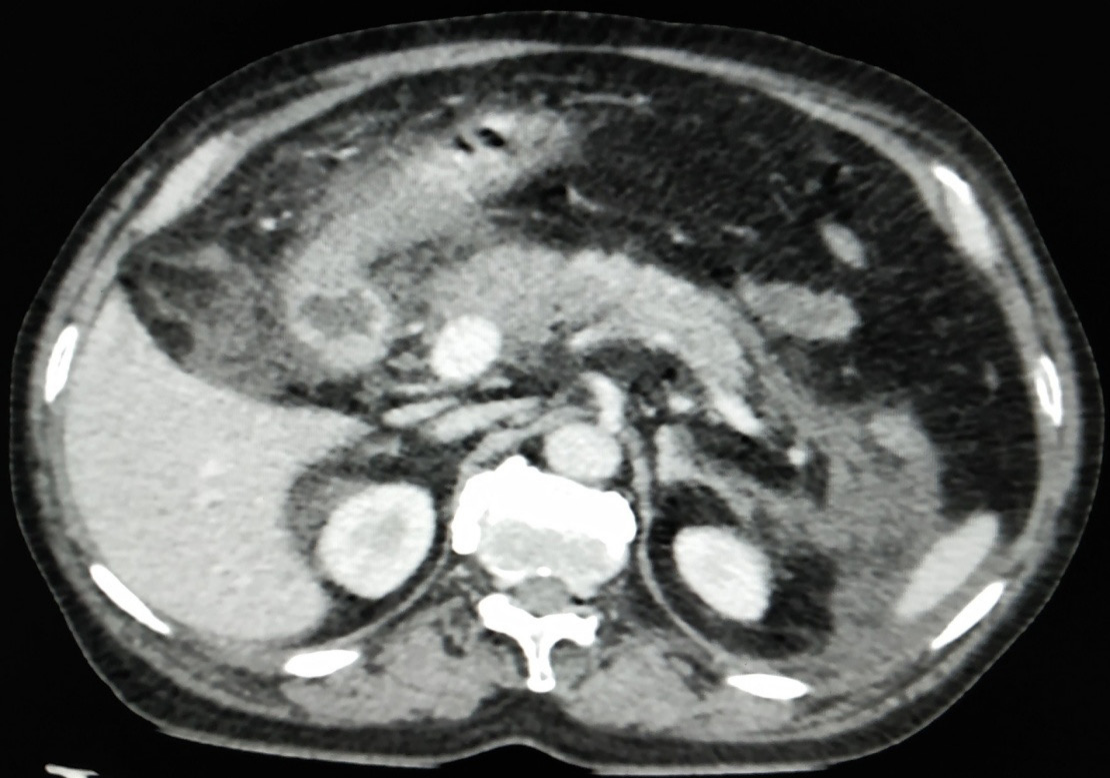
Contrast enhanced computed tomography of abdomen shows enlarged tail, distal part of body with indistinct margin, normal enhancement and peritoneal collection.

A chest x-ray showed right-sided middle zone infiltrates. Ultrasound of the abdomen showed the bulky head of the pancreas. Contrast-enhanced computed tomography (CT) of the abdomen showed normal enhancing pancreas with swollen distal part of body and tail region with indistinct margin, peripancreatic inflammation, fluid collection in peritoneal cavity and bilateral pleural effusion. The modified CT severity index was 6 (Figure 1).

His investigation profiles were total leucocyte count (TLC)-21000/mm^3^, platelets-90000/mm^3^, hemoglobin (Hb)-9gm/dl, urea 135mg/dl, creatinine 1.8mg/dl. Sodium and potassium were 125mmol/L and 5.1mmol/L respectively. Liver function test (LFT) showed bilirubin-2.5mg/dl; Direct bilirubin 1.8mg/dl;Total protein 7.8mg/dl;albumin 4.5mg/dl;alanine aminotransferase (ALT)-402U/L and aspartate aminotransferase(AST)-501U/L Random blood sugar was 561mg/dl and urine acetone + + + +. Serum amylase and lipase were 2346U/L and 1256U/L respectively.

The patient was diagnosed as Acute severe alcoholic pancreatitis; Diabetic ketoacidosis precipitated by right side community-acquired pneumonia; septic shock with multi-organ dysfunction. Resuscitation was continued with balanced salt solution and noradrenaline. He was started on Piperacillin-tazobactam 4.5gram intravenous every six hours (Aristo Pharmaceutical Pvt Ltd,Manideep, India) and Doxycycline 100mgintravenous every twelve hours(Gufic Biolifesciences, Mumbai, India), Regular insulin 6U intravenous was given and Regular Insulin was started as per ICU protocol. He was also started on Ulinastatin 2 million units intravenous every twelve hours (Bharat Serum, Mumbai, India) in 100ml normal saline over 1 hour for 5 days.

Noradrenaline was stopped on the third day, serum creatinine, LFT, TLC , blood sugar was monitored daily and was noted to be in improving trend until it was normal on the third day of admission. The patient also improved clinically there was a decrease in abdominal distension, tolerance of feeding by the fifth day. He was discharged from ICU on the sixth day and from the hospital on a ninth day.

One week following discharge, he presented at the outpatient for a follow-up. He was not having any symptoms and was advised to have followed up monthly.

### CASE 2

A 36-year-old male, weighing 130kilograms with a history of type 2 diabetes mellitus, obstructive sleep apnea and hypertension under the irregular medication and daily consumer of one bottle of alcohol presented at the Emergency department of a general super specialty hospital, Birtamod, Nepal. He presented with a history of diffuse abdominal pain, 10/10 in severity, aggravated by deep breathing and relieved by leaning forward and multiple episodes of non-projectile, non-bloody vomiting. He also complained of high-grade fever and burning micturition for 5 days.

At the time of admission to the emergency department his GCS was 15/15, pulse rate 126 beats per min, blood pressure 60/40mm Hg, respiratory rate 34/min and oxygenation saturation was 92% on 10liters of oxygen. The chest examination was normal. Abdominal examination showed tenderness, guarding and rigidity at the epigastric region. Arterial blood gas analysis showed high anion gap metabolic acidosis and lactic acidosis.

An emergency physician and paramedics immediately resuscitated him by inserting 16 gauze cannula, central venous catheter and giving two liters of balanced salt solution Kabilyte (Fresenius Kabi, Pune, India) and noradrenalin was started at 0.3yyg/kg/min.

**Figure 2:**
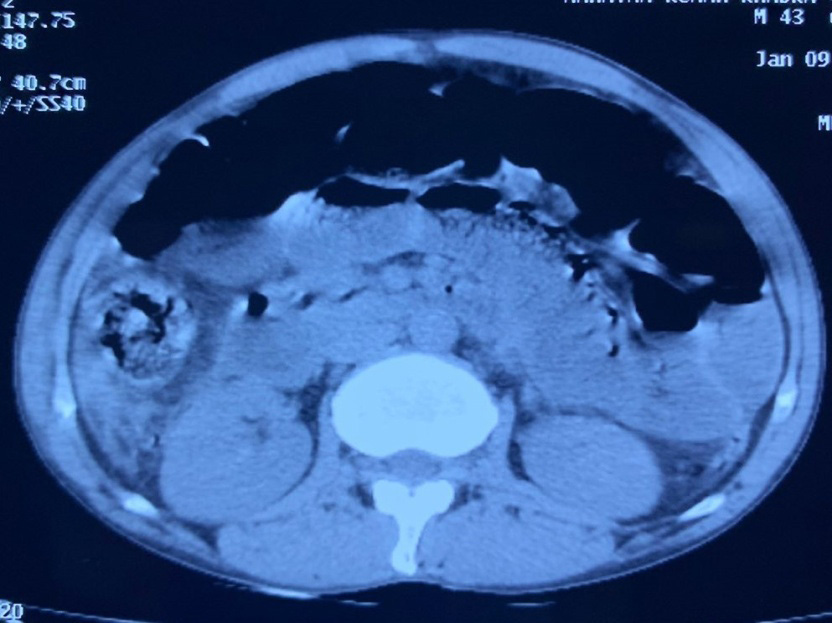
Contrast enhanced computed tomography of abdomen shows enlarged head, body with indistinct margin, normal enhancement and peritoneal collection.

Ultrasound of the abdomen showed the bulky head and neck of the pancreas. The plain x-ray of the abdomen was normal. Contrast-enhanced computed tomography (CT) of the abdomen showed normal enhancing pancreas with swollen head, body peripancreatic inflammation, fluid collection. The modified CT severity index was 6 (Figure 2).

His investigation profiles were total leucocyte count (TLC)-19200/mm^3^, platelets-60000/mm^3^, hemoglobin(Hb)-9gm/dl, urea 135mg/dl, creatinine 2.2mg/dl. Sodium and potassium were 131mmol/L and 4.9mmol/L respectively. Liver function test (LFT) showed bilirubin-3.0mg/dl; Direct bilirubin 1.6 mg/dl;Total protein 7.8mg/dl;albumin 5.4mg/dl;alanine aminotransferase (ALT)-561U/L and aspartate aminotransferase(AST)-798U/L Random blood sugar was 456mg/dl and urine acetone + + + +,sugar + + + and pus cell plenty. Serum amylase and lipase were 1956U/L and 1102U/L respectively.

The patient was diagnosed as Acute severe alcoholic pancreatitis; diabetic ketoacidosis precipitated by complicated urinary tract infection; septic shock with multi-organ dysfunction. Resuscitation was continued with balanced salt solution and noradrenaline. He was started on Piperacillin-tazobactam 4.5gram intravenous every six hours (Aristo Pharmaceutical Pvt Ltd,Manideep, India) and Doxycycline 100mg intravenous every twelve hours (Gufic Biolifesciences, Mumbai, India), Regular insulin 6U intravenous was given and Regular Insulin was started as per ICU protocol. He was also started on Ulinastatin 2 million units intravenous every twelve hours (Bharat Serum, Mumbai, India) in 100ml normal saline over 1 hour for 5 days.

Noradrenaline was stopped on the fourth day, serum creatinine, LFT, TLC , blood sugar was monitored daily and was noted to be in improving trend until it was normal on the fifth day of admission. The patient also improved clinically there was a decrease in abdominal distension, tolerance of feeding by the fourth day. He was discharged from ICU on the sixth day and from the hospital on an eighth day.

One week following discharge, he presented at the outpatient for a follow-up. He was not having any symptoms and was advised to have followed up monthly.

### CASE 3

A 76-year-old female, a history of type 2 diabetes mellitus, chronic obstructive airway disease obstructive sleep apnea and hypertension under the irregular medication and daily consumer of one bottle of alcohol; 10 pack-year of smoking presented at the Emergency department of a general super specialty hospital, Birtamod, Nepal. He presented with a history of epigastric abdominal pain, 8/10 in severity, aggravated by deep breathing and relieved by leaning forward and multiple episodes of non-projectile, non-bloody vomiting. She also complained of high-grade fever, productive, non-foul smelling, yellowish cough and shortness of the breath exacerbated by cough and has increased in severity from grade 2 to 4.

At the time of admission to the emergency department his GCS was 10/15, pulse rate 110 beats per min, blood pressure 90/60mm Hg, respiratory rate 34/min and oxygenation saturation was 92% on 15liters of oxygen. The chest examination showed bilateral wheeze and crepitation. Abdominal examination showed tenderness, guarding at the epigastric region. Arterial blood gas analysis showed high anion gap metabolic acidosis, respiratory acidosis and lactic acidosis.

An emergency physician and paramedics immediately resuscitated him by inserting 16 gauze cannula, central venous catheter and giving two liters of balanced salt solution Kabilyte (Fresenius Kabi, Pune, India) and noradrenaline was started at 0.1μg/kg/min.

**Figure 3:**
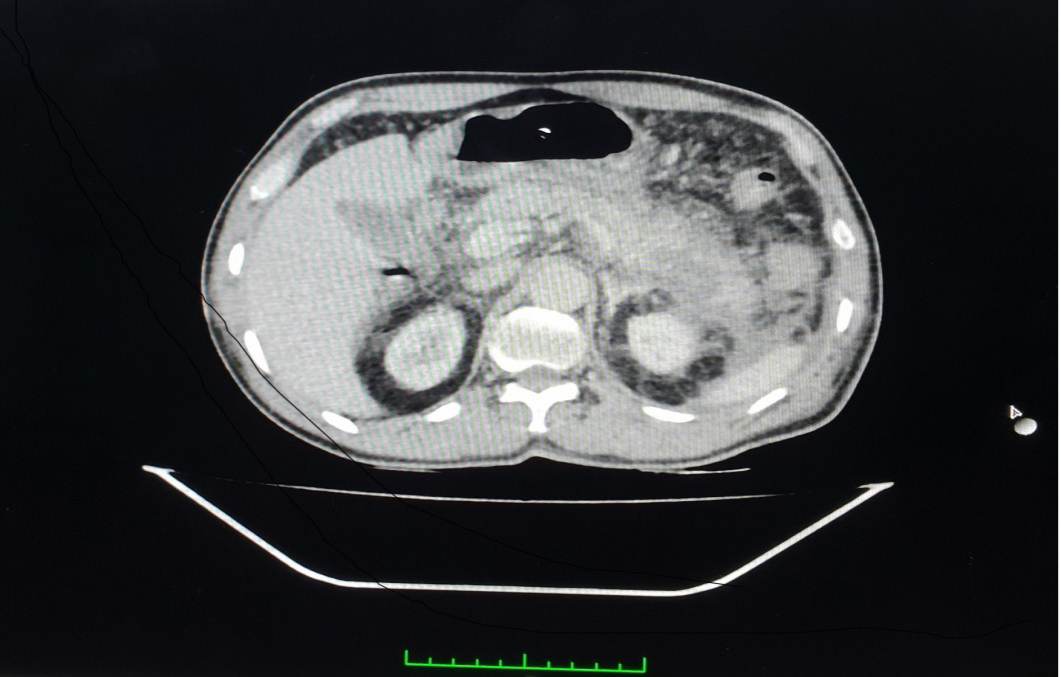
Contrast-enhanced computed tomography of abdomen shows pancreatic necrosis in 30% with swollen neck and tail, peripancreatic inflammation and fluid collection.

A chest x-ray showed bilateral lower zone pneumonia, Ultrasound of the abdomen showed the bulky neck and tail of the pancreas. The plain x-ray of the abdomen was normal. Contrast-enhanced computed tomography (CT) of the abdomen showed pancreatic necrosis in 30% with swollen neckand tail, peripancreatic inflammation, fluid collection and bilateral pleural effusion. The modified CT severity index was 8 (Figure 3).

His investigation profiles were total leucocyte count (TLC)-29200/mm3, platelets-48000/mm3, hemoglobin (Hb)-13gm/dl, urea 135mg/dl, creatinine 1.6mg/dl. Sodium and potassium were 138 mmol/L and 4.9mmol/L respectively. Liver function test (LFT) showed bilirubin- 2.8mg/dl; Direct bilirubin 1.6mg/dl;Total protein 5.4mg/dl; albumin 3.4mg/dl; alanine aminotransferase (ALT)- 412U/L and aspartate aminotransferase (AST)-1012U/L Serum amylase and lipase were 1956U/L and 1102U/L respectively.

The patient was diagnosed as Acute severe alcoholic pancreatitis; Acute exacerbation of chronic obstructive airway disease with type 2 respiratory failure precipitated by bilateral pneumonia; septic shock with multi-organ dysfunction. Resuscitation was continued with balanced salt solution and noradrenaline. He was started on Piperacillin-tazobactam 4.5gram intravenous every six hours (Aristo Pharmaceutical Pvt Ltd, Manideep, India) and Doxycycline 100mg intravenous every twelve hours(Gufic Biolifesciences, Mumbai, India), Bilevel positive airway pressure (BiPAP) . He was also started on Ulinastatin 2 million units intravenous every twelve hours (Bharat Serum, Mumbai, India) in 100 ml normal saline over 1 hour for 5 days.

Noradrenaline was stopped on the fifth day, serum creatinine, LFT, TLC; arterial blood gas was monitored daily and was noted to be in improving trend until it was normal on the ninth day of admission. The patient also improved clinically there was a decrease in abdominal distension, tolerance of feeding, cough and shortness of breath by the fifth day. He was discharged from ICU on the fifth day and from the hospital on an eighth day.

One week following discharge, he presented at the outpatient for a follow-up. He was not having any symptoms and was advised to have followed up monthly.

## DISCUSSION

Severe acute pancreatitis is defined by modified atlanta's classification as organ failure that persists for greater than 48 hours.^3,4^ Organ failure can be single or multiple and is defined by modified marshall scoring system. ^5^ All of our patients had hypotension and acute kidney injury that resolved in 3-6 days without any complications while a study by Bhattarai, et al.^6^ has shown that respiratory complication is more common than other complication. This difference may due to that all patient had diabetes; urinary tract infection is more than pneumonia in diabetes, different patient population.

Studies have shown that gall stone pancreatitis is more common than alcoholic pancreatitis but our all patient were alcoholic. This difference may due to the high prevalence of alcohol intake in this eastern region of Nepal.

Premature activation of digestive enzymes within the acinar cells of the pancreas leads to auto-digestion of the pancreas and early inflammatory reaction within the pancreas with neutrophil and monocyte leads to damage to the pancreas and mortality in the first week of pancreatitis.

Management of pancreatitis is supportive; with the unavailability of drugs that can decrease digestion and inflammation of the pancreas. Ulinastatin is a serine proteases inhibitor that decreases the inflammation and dysregulated coagulation.^7^ It has also reversed histological damage and inflammation in the pancreas in the experimental models of severe acute pancreatitis.^8^ Studies have shown that ulinastatin decreases the C-reactive protein, interleukin-6 and tumor necrosis factor.^7^ It also decreases the organ dysfunction, the local complication of pancreatitis. Studies have shown that ulinastatin decreases T-cell apoptosis and tissue damage by modulating regulatory T-cell^8^ that leads to a decrease in mortality that was observed in our patients.

Ulinastatin has shown to be effective in idiopathic and endoscopic retrograde cholangiopancreatography induced pancreatitis^11^ and severe pancreatitis.^6,7^ Therefore, it can be effective in alcohol-induced pancreatitis, large studies are required to evaluate the effectiveness of ulinastatin in alcohol-induced pancreatitis.

Ulinastatin can be used alone or in combination with other protease inhibitors like aprotinin, nafamostat mesilate and gabexate mesilate^6,12,13^ and somatostatin^14^ for severe pancreatitis but ulinastatin was only used in our patients due to the unavailability of other protease inhibitors.

Studies have shown that ulinastatin can also be given an intra-arterial continuous infusion to decrease the mortality in necrotizing pancreatitis^15^ but it was given intravenously over 1-hour infusion in our patients.

Severe acute pancreatitis has high mortality but newer drugs like anti-proteases inhibitor, somatostatin, vitamin C, glutamine, albumin, thymopentin^12^ can be used in combination or alone to decrease the mortality. Guidelines from china and japan have recommended early use of anti-protease inhibitor^13^ but large scale studies are required to know the effectiveness and mode of administration of these drugs.

Ulinastatin should be started early in severe acute alcoholic pancreatitis that can improve patient outcome but a large randomized controlled trial is required to confirm this.
